# A Feasibility Study of Real-Time FMRI with Neurofeedback of Motor Performance in Cerebellar Ataxia

**DOI:** 10.3390/brainsci16020120

**Published:** 2026-01-23

**Authors:** Joshua G. Berenbaum, Cherie L. Marvel, Jonathan M. Lisinski, Jeffrey S. Soldate, Owen P. Morgan, Ashley N. Kucharski, Luca P. Lutzel, Jonathan A. Ecker, Laura C. Rice, Amy Mistri, Prianca A. Nadkarni, Liana S. Rosenthal, Stephen M. LaConte

**Affiliations:** 1 Department of Neurology, Johns Hopkins University School of Medicine, Baltimore, MD 21287, USA; jberenb1@bu.edu (J.G.B.); opm6@cornell.edu (O.P.M.); akuchar4@jh.edu (A.N.K.); amymistri@gmail.com (A.M.); liana.rosenthal@jhmi.edu (L.S.R.); 2 Fralin Biomedical Research Institute at VTC, Virginia Tech, Roanoke, VA 24016, USA; jlisinski@vtc.vt.edu (J.M.L.); jsoldate@vtc.vt.edu (J.S.S.); 3 Department of Biomedical Engineering, Virginia Tech, Blacksburg, VA 24061, USA; 4 Center for Neurodevelopmental and Imaging Research, Kennedy Krieger Institute, Baltimore, MD 21205, USA; laura.c.rice.phd@gmail.com

**Keywords:** spinocerebellar ataxia, movement disorder, neurofeedback, cerebellum, motor function, functional MRI, real-time fMRI

## Abstract

Background/Objectives: Neurodegenerative cerebellar ataxia (CA) is a movement disorder caused by progressive cell death in the cerebellum. Motor imagery represents a potential therapeutic tool to improve motor function by “exercising” brain regions associated with movement, without the need for overt activity. This study assessed the feasibility of combining motor imagery with real-time functional magnetic resonance imaging neurofeedback (rt-fMRI-NF) to improve motor function in CA. Methods: During finger tapping conditions, 16 participants with CA pushed a button at the same frequency in time with cross flashing at 1 Hz or 4 Hz, and this information was used to train the model. During motor imagery, participants imagined finger tapping while undergoing rt-fMRI-NF with visual feedback, steering them toward activating their motor circuit. Afterwards, they completed finger tapping again. FMRI analysis compared successful motor imagery trials versus all other imagery events. Brain activity on successful trials was covaried with pre–post rt-fMRI-NF tapping improvement scores. Results: Tapping was more accurate at 1 Hz than 4 Hz, and larger tapping error rates correlated with greater movement impairments. While not significant at the group level, 9 of the 16 participants improved tapping accuracy following rt-fMRI-NF. The size of motor improvements correlated with successful motor imagery activity at 1 Hz in the frontal lobe, insula, parietal lobe, basal ganglia, and cerebellum. Motor improvements were not associated with neurological impairment severity, mood, cognition, or imagery vividness. Conclusions: Feasibility was demonstrated for motor imagery therapy with neurofeedback to potentially improve fine motor precision in people with CA. Brain regions relevant to this process may be considered for targets of non-invasive therapeutic interventions.

## 1. Introduction

Neurodegenerative cerebellar ataxia (CA) is a specific type of ataxia that results from progressive degeneration of the cerebellum, commonly caused by genetic factors [1,2]. The cerebellum coordinates movement with other brain motor regions and similarly contributes to mood and cognition, including executive function and working memory [3,4,5,6,7,8,9]. Therefore, CA presents with balance and gait difficulties, problems with speech and swallowing, loss of eye movement control, impaired vision, mood changes, and impaired cognitive abilities, which can greatly impact quality of life [10]. No therapies or medications exist to cure CA’s underlying neurodegeneration, and care predominantly targets amelioration of symptoms. Nonpharmacologic strategies include exercise and physical, speech, and swallowing therapy [11,12,13,14]. Pharmacologic treatments like baclofen and gabapentin are commonly used for nystagmus [2]. However, these approaches have limits; therapy can be difficult or have unacceptable side effects, and medications often provide variable, incomplete benefit [2].

Motor imagery is a promising, low-risk adjunct for treatment of symptoms in CA and other movement disorders. When combined with real-time fMRI neurofeedback (rt-fMRI-NF), participants receive feedback on their imagery-evoked brain activity [15]. In healthy individuals, motor imagery engages many of the same motor regions as motor execution, including the premotor cortex, supplementary motor area (SMA), basal ganglia, cerebellum, and inferior parietal lobes [16,17,18,19]. Furthermore, motor imagery can activate cognitive and visual areas, such as the frontal and occipital lobes [20,21,22]. With neurofeedback, individuals have learned to volitionally modulate these motor imagery activations [23,24,25], which may strengthen relevant networks and support functional gains [26]. Emerging studies support the clinical promise of guided motor imagery. For example, patients with Parkinson’s disease improved motor control with imagery plus virtual reality [27], and stroke patients showed functional gains with imagery and NIRS-mediated neurofeedback [28].

When utilizing rt-fMRI-NF during motor imagery, healthy and clinical populations (e.g., diagnosed with stroke or Parkinson’s disease) have self-regulated brain activation levels and consequently improve motor control and coordination by strengthening these brain pathways [26,29,30,31,32,33,34]. In healthy participants, imagery-driven up-regulation of the sensorimotor cortex improved performance [33,34], and in Parkinson’s disease, SMA-targeted feedback enhanced finger-tapping [29]. These findings support further evaluation of rt-fMRI-NF as an adjunct treatment in CA.

The present study investigated whether a motor imagery approach, in conjunction with rt-fMRI-NF, could improve motor function in CA by “exercising” participants’ neural imagery pathways. A finger tapping task was used as the performance measure before and after the rt-fMRI-NF intervention. It was hypothesized that rt-fMRI-NF would lead to increased precision in tapping task performance. A finger tapping task was chosen due to its being conducive to the MRI environment. Importantly, finger movements represented fine motor impairments of CA, and improvement at this smaller level could provide proof of concept for the application of motor imagery therapy methods to more complex movements, such as limb and gait function.

## 2. Materials and Methods

### 2.1. Participants

Twenty-one participants with neurodegenerative cerebellar ataxia (CA) were recruited for this non-randomized single-arm before–after clinical trial (ClinicalTrials.gov (https://clinicaltrials.gov/study/NCT05436262, accessed on 17 December 2024) registered 23 June 2022). Recruitment was performed through the ataxia clinic at the Johns Hopkins Outpatient Center, the ClinicalTrials.gov website, and the National Ataxia Foundation newsletter. The study was approved by the Institutional Review Board of Johns Hopkins University School of Medicine and was conducted in accordance with the Declaration of Helsinki. Informed consent was obtained, and participants were compensated for their participation. Participants were included if they had been diagnosed with progressive cerebellar degeneration and other causes, such as stroke, virus/bacteria, or environmental factors, had been ruled out. Exclusion criteria included having a (1) diagnosis of a major psychotic disorder; (2) substance use disorder; (3) neurologic disorder aside from ataxia (e.g., epilepsy, autism, or attention deficit hyperactivity disorder); (4) stroke or other damage to the brain unrelated to CA; (5) self-reported history of a learning disability; or (6) head injury with loss of consciousness longer than 5 min. Additionally, all participants in the study were required to be right-handed. CA diagnoses were verified by a trained movement disorders neurologist (LSR) who categorized cases of spinocerebellar ataxia (SCA) into known subtypes or otherwise as cerebellar ataxia of unknown etiology (CAUE) based on information from patients’ health histories, clinical examination data, genetic testing, and neuroimaging. Genetic testing documenting the patients’ diagnosis was provided upon arrival for the study.

One ataxia participant could not complete the MRI scan due to extreme dizziness when they reclined to a supine position, which was a specific symptom related to their CA. Based on post-experimental questionnaires describing the motor imagery strategies used during real-time fMRI, data from four participants with unacceptable strategies (as described in Section 2.3.3) were removed from analyses for a total of sixteen participants. Table 1 lists demographic information for included participants (See Table S1 for demographic information of excluded participants and reported strategies).

### 2.2. Clinical and Cognitive Assessments

We administered a series of cognitive and behavioral tests, in the same ordered sequence for each participant: (1) the Hamilton Anxiety Scale (HAM-A) [35]; (2) Hamilton Depression Rating Scale (HAM-D) [36]; (3) Wide-Range Achievement Test 4 (WRAT4) [37]; (4) Cerebellar Cognitive-Affective Syndrome (CCAS) Scale [38]; (5) Kinesthetic and Visual Imagery Questionnaire 10 (KVIQ-10) [39]; (6) Ataxia Assessment of Function (AAF) [10]; and (7) the International Cooperative Ataxia Rating Scale (ICARS) [40]. ICARS was administered, video-recorded, and scored by consensus (Table 1).

### 2.3. MRI Data Acquisition

All MRI data were acquired using a 3.0T Philips Elition Scanner, using a 32-channel head coil. A sagittal magnetization-prepared gradient-echo (MPRAGE) sequence aligned to the anterior–posterior commissure (AC-PC) axis was used with the following parameters: TR = 3000 ms; TE = 3.3 ms; TI = 985 ms; field of view = 240 mm × 240 mm; 170 slices; slice thickness 1.0 mm; 0 mm gap; flip angle = 8°; voxel size = 0.75 mm × 0.75 mm; and SENSE = 2 (6 min). For six functional scans (i.e., “runs”), a T2-weighted gradient echo EPI pulse sequence was used with the following parameters: TR = 2000 ms; TE = 30 ms; flip angle = 61 degrees; in-plane resolution = 3.5 mm; slice thickness = 4.0 mm; 0 mm gap; 39 oblique-axial slices aligned along the AC-PC axis; and FOV = 224 mm × 224 mm. The fMRI scan triggered the stimulus presentation at the beginning of each run (7 min/run).

#### 2.3.1. MRI Equipment and rt-fMRI

Stimuli were delivered using the Python Vision Egg library (1.2.1) on a Dell Inspiron 7472 laptop running Windows 10 Pro [41]. Participants viewed stimuli via a Cambridge Research Systems BOLDscreen 32 UHD LCD display, connected to a mirror box for a right–left image flip, then projected onto the head coil-mounted mirror. Button-press responses were collected using two fiber optic button boxes (MRA, Inc., Washington, PA, USA) held by participants in their right hand.

rt-fMRI-NF was performed using online whole-brain classifiers, following our previous methods [42,43,44,45]. Briefly, TCP/IP communication transmitted images to a Linux workstation, running AFNI’s real-time plugin [46,47] version 21.2.05, with modifications specific to this experiment at https://github.com/lacontelab/afni_rt_philips/, accessed on 2 January 2024, and the AFNI tool, 3dsvm [48]. Classifier output from 3dsvm was then transmitted by TCP/IP to Vision Egg . In previous studies on Siemens systems, our scanner-to-AFNI TCP/IP transmission was compiled into image reconstruction software. For this study, however, we used in-house software to monitor reconstructed fMRI images as they were written to disk. In total, this resulted in a 4 s latency between volume acquisition and AFNI’s data reception.

**Figure 1:**
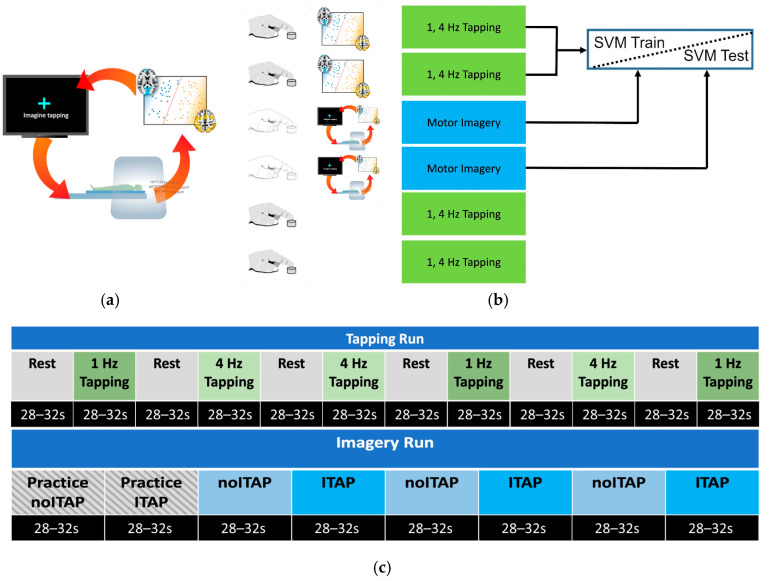
Study design. For each participant, a whole-brain SVM classifier of tapping versus rest was trained. (**a**) During rt-fMRI, as a participant performed motor imagery or non-motor imagery, MRI volumes were classified, and performance feedback (e.g., flashing cross) was provided based on the distance to the hyperplane. This steered participants to control on/off activation of motor brain regions. (**b**) MRI runs were divided into overt tapping and motor imagery. (**c**) Tapping and imagery runs broken down by block. Each tapping block presented a flashing cross at one of two speeds: 1 Hz or 4 Hz. Imagery blocks alternated between Imagine Tapping (ITAP) and Do Not Imagine Tapping (noITAP) blocks, in which a cross was presented as static or flashing, and participants were instructed to make the cross flash (ITAP) or stop flashing (noITAP), respectively, using mental imagery. Two practice blocks were presented at the beginning of each imagery run, imitating one noITAP block followed by one ITAP block with ideal feedback performance.

The general strategy for classifier-based rt-fMRI-NF was to first train a classifier on initial fMRI data and then use that classifier to decode new fMRI volumes online. The volume-by-volume classification serves as a control signal to update the stimulus display (Figure 1a). Here, participants performed two tasks across six runs (Figure 1b). The first two runs were classifier training runs, consisting of a finger tapping task (described below). Subsequently, two runs of motor imagery (described below) were used for rt-fMRI-NF. Finally, two additional runs of the finger tapping task were collected to examine classifier performance and assess any changes in motor performance between the beginning and end of the session (Figure 1c). To this end, a group mask was generated based on the MNI T1 template using 3dAutomask within AFNI [49]. Due to anticipated visual cortex activity elicited by the flashing cross during tapping and motor imagery tasks, a visual cortex mask was used to exclude primary visual areas from the support vector machine (SVM) model. The visual cortex mask was created by forming a union of five specific cytoarchitectonic regions (Area 17 (V1), Area 18 (V2), hOC3v, hOC4v, and hOC5 (V5/MT)) extracted from the Eickhoff–Zilles atlas within AFNI [50,51,52,53,54]. This mask was then resampled to the EPI grid, retaining any voxel that occupied at least 20% of the volume. A linear SVM classifier was trained to differentiate between tapping and rest during the first two tapping runs using the mask above. Training was performed offline between the second tapping run and the first imagery run on data we had corrected for slice timing, motion, baseline, and linear trends, and blurred to 6mm FWHM. Data was prepared in real-time for prediction with similar pre-processing, with regression of motion and linear trends performed using data from the volumes collected prior to the most recent acquisition within the same run.

#### 2.3.2. FMRI Tapping Task

The tapping task was used to establish the classifier model (2-class: tap vs. rest) for the motor imagery neurofeedback. Each run contained six blocks of tapping (either 1 Hz or 4 Hz) that alternated with six blocks of rest (Figure 1c). The duration of each block was 28–32 s. All subjects received the same order as shown in Figure 1c. Participants used an MR-compatible button box (MRA, Inc., Washington, PA, USA) and were instructed to use their right index finger to tap in time with a flashing blue cross on a screen in front of them (following procedures in [55]). The task was explained to participants during a simplified pre-MRI practice session, which familiarized participants with the button box and the gray feedback bar used in the tapping blocks. As in our prior studies [55,56], a gray feedback bar indicated the most recent tap-to-tap rate based on its right–left location and, thus, whether the last press was slower than, in time with, or faster than the flashing speed. For rest blocks, participants viewed a blue non-flashing cross on a black background (See Video S1).

Participant tapping behavior was quantified by measuring the instantaneous tapping rate during the task. For each new tap, the time elapsed since the last tap was measured, converted to a rate, and then compared to the instructed rate. The error was the deviation of the instantaneous rate for each tap from the instructed rate (1 Hz or 4 Hz). Each run was scored based on the root mean square error (RMSE), which decreased across all blocks. Prolonged periods of inactivity were identified if the inter-tap interval exceeded the instructed rate. This was quantified as the number of missed taps/number of ideal taps.

#### 2.3.3. FMRI Imagery Task

The imagery task consisted of two block conditions: Imagine Tapping (ITAP) and Do Not Imagine Tapping (noITAP), as shown in Figure 1c. Each imagery run began with two practice blocks, followed by six alternating blocks of motor imagery (Imagine Tapping, ITAP) and non-motor imagery (Do Not Imagine Tapping, noITAP) (See Videos S2 and S3). At the beginning of each rt-fMRI-NF imagery run, two “Practice” blocks were presented, which displayed the ideal performance of the cross-flashing behavior (as if participants perfectly modulated their motor region activity in the ITAP and noITAP blocks). Participants were asked to work hard on all blocks, including practice blocks. The practice blocks were used to kick-start data analysis to improve neurofeedback accuracy. By having both an initial ITAP and a noITAP block, fMRI data pre-processing could begin to correct for motion, linear detrending, and any classifier-to-data mismatches.

During the pre-MRI practice session, participants were instructed to use mental imagery during ITAP blocks to imagine themselves tapping and verbalize to the experimenter a strategy to use in the MRI. They were informed that during ITAP in the MRI a white static cross would turn blue and begin to flash if they were robustly activating their motor circuit. If they could not get the cross to flash, they were instructed to change strategies to make their imagery more vivid or otherwise imagine tapping more actively. They were instructed to try different strategies until successful motor imagery was achieved, which occurred when brain regions consistent with the neurofeedback model were activated. If so, the cross flashed at 2 Hz. During the noITAP blocks, a flashing cross (2 Hz) appeared on a black screen, and participants were instructed to make the cross stop flashing using non-motor imagery (e.g., viewing a sunset), which disengaged motor-related brain activity.

Participants were informed that a good mental imagery strategy was necessary for the rt-fMRI-NF imagery task to function. During ITAP blocks, acceptable strategies were motor-focused. For instance, one participant reported that imagining “pounding a baseball glove” was most effective. Unacceptable ITAP strategies consisted of non-motor thought processes such as “willing” the cross to flash (i.e., some form of believed telekinesis) or overt movements that reflected actual motor activity rather than imagery. Overt movements were assessed via experimenter observations rather than objective measures, and small, unintentional movements during motor imagery may have occurred. During noITAP blocks, acceptable strategies were thought up through processes that involved non-motor imagery. One participant accomplished this by “[imagining that I was] lying on the beach, listening to the waves.” Unacceptable noITAP strategies consisted of motor imagery or overt movements. The neurofeedback aspects of the imagery task, therefore, steered participants toward strategies that modulated the flashing cross, which, in turn, trained participants to self-modulate their motor circuit.

Following data collection (offline), classifier performance was measured on each TR as a true positive (flashed when it was supposed to), false positive (flashed when it was not supposed to), true negative (stopped flashing when it was supposed to), and false negative (stopped flashing when it was not supposed to). ITAP accuracy was defined by the number of true positive TRs (i.e., “true ITAP”), divided by the total ITAP TRs (true ITAP/all ITAP). Similarly, noITAP was defined by true negative TRs divided by the total noITAP TRs (i.e., “true noITAP”/all noITAP). Overall imagery accuracy was defined by (true ITAP + true noITAP)/(all ITAP + all noITAP).

### 2.4. Post-FMRI Questionnaire

A post-fMRI questionnaire assessed imagery strategies, using open-ended questions. Participants noted their perceived optimal strategy for both making the cross flash and for making the cross not flash. Post-session consensus ratings (evaluated by authors C.L.M. and L.P.L.) determined whether the participants’ strategies were considered acceptable or unacceptable for data inclusion.

### 2.5. FMRI Data Analysis

#### 2.5.1. Whole-Brain Analysis

Standard image processing steps were performed using SPM12 [57], including slice timing (reference slice = 20), motion correction (aligned to first volume of first run), co-registration of the anatomical to functional images, normalization to Montreal Neurological Institute (MNI) stereotaxic space, and spatial smoothing (FWHM = 8 mm). Individual statistical maps were computed for each subject using the GLM approach. Canonical hemodynamic response function (HRF) regressors were convolved with tapping blocks at 1 Hz, 4 Hz, and rest (block design: Runs 1, 2, 5, 6), with successful motor imagery events (true ITAP), and with all other imagery events (true noITAP, false positives, and false negatives; event-related design: Runs 3 and 4). A random effects analysis was then performed to map the mean blood oxygen level dependence (BOLD) responses. For tapping runs, contrasts were created for tapping at each speed minus rest. For motor imagery runs, contrasts were created for successful motor imagery events minus all other events. Beta contrast volumes per subject were used to conduct one-sample *t*-test values at every voxel to reveal activation clusters related to tapping and successful motor imagery. For motor imagery, a covariate analysis was also conducted on motor imagery runs that entered the tapping improvement scores (delta (Δ) root mean square mean error (RMSE) between tapping Runs 2 and 5 at 1 Hz and 4 Hz), controlling for ICARS scores, to reveal activation clusters that increased or decreased with tapping improvements. Activation thresholds were set to *p* < 0.001 and SPM12’s minimum expected voxels per cluster, which were k = 17 for tapping, k = 16 for motor imagery, and k = 15 with covariates.

For anatomical determinations of the activations, MNI coordinates were transformed into the coordinate system of the Talairach and Tourneaux stereotaxic atlas [58] using Bioimage Suite Web (Version 1.2.0) [59] and cross-referenced with atlas manuals [58,60]. Regions of interest (ROIs) were created from clusters that survived the covariate analysis. MRI values were computed from these ROIs using the Mars-BaR toolbox [61] for SPM and correlated with tapping improvement scores to create scatterplots for visual inspection of the data.

#### 2.5.2. Cerebellar Analysis

Given that cerebellar atrophy is associated with CA, functional activations were further investigated using the Spatially Unbiased Atlas Template (SUIT) of the human cerebellum [62] (www.diedrichsenlab.org/imaging/suit_fMRI.htm, accessed on 25 March 2025). Using functional images that had been pre-processed for slice-timing correction and realignment, images were co-registered to the anatomical image. Individual statistical maps were computed for each subject using the GLM approach, as described above, yielding beta contrast volumes for each participant. The anatomical image was entered into the SUIT toolbox (v3.7) to isolate the cerebellum and brainstem from the rest of the brain and to segment tissues. Probabilistic maps were normalized into SUIT space using diffeomorphic anatomical registration through the exponentiated lie (DARTEL) algebra algorithm [63]. This deformation was applied to contrast images from the first-level analysis by reslicing them into SUIT space. Finally, images were smoothed using a 4 mm isotropic kernel due to the small lobules within cerebellar anatomy. Second-level group analyses were conducted, including a mask of the cerebellum and brainstem generated from the SUIT to avoid activation influences from the inferior occipital cortex according to standard implementation of the SUIT analysis pipeline [64]. Results were generated using SPM12. Thresholds were set to *p* < 0.001 and SPM12’s minimum expected voxels per cluster: k = 12 for tapping, k = 11 for motor imagery, and k = 10 with covariates. All cerebellar-related data reports were based on the SUIT analysis.

### 2.6. Statistics

Demographic, clinical, cognitive, and MRI data contained continuous variables, except gender, which was categorical. For group comparisons of those included versus excluded from the dataset, Mann–Whitney *t*-tests were used, and Fisher’s Exact test was used to compare gender. RMSE values for tapping and percent accuracy for imagery were subjected to repeated-measures ANOVAs to determine change across blocks. Mauchly’s Test of Sphericity was applied, and if the assumption of sphericity was violated, a Greenhouse–Geisser correction was used to adjust degrees of freedom. Wilcoxon Signed-Rank *t*-tests were used for tapping blocks when RMSE was compared for paired samples between Runs 2 and 5 at 1 Hz and 4 Hz. Spearman’s rho nonparametric correlations compared MRI ROI values of successful images with improvement in the tapping task, as well as associations between improvement scores and clinical and cognitive assessments. Significant correlations were subjected to visual inspection of scatterplots. All tests were two-tailed, with an alpha level < 0.05 to define statistical significance. Statistics were performed using IBM SPSS Statistics, Macintosh, version 29 (IBM Corp., Armonk, NY, USA).

## 3. Results

### 3.1. FMRI Task Performance

#### 3.1.1. Tapping Task

**Figure 2:**
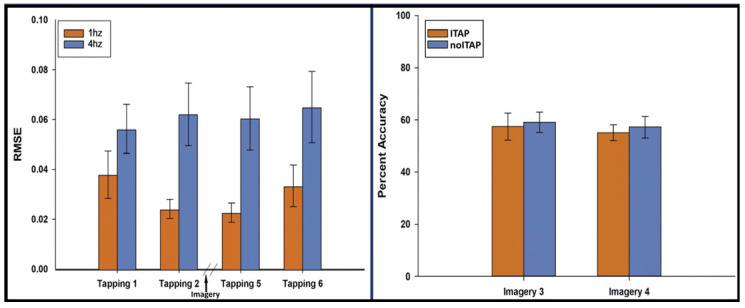
Performance on the tapping and imagery tasks. (**left**) RMSE of tapping for Runs 1, 2, 5 and 6; (**right**) imagery accuracy for ITAP and noITAP for Runs 3 and 4.

Performance was measured as the RMSE of the deviation from the actual tap rate to the instructed rate. At 1 Hz, the mean RMSE (SD) was 0.038 Hz (SD = 0.038) for Run 1, 0.024 Hz (SD = 0.015) for Run 2, 0.023 Hz (SD = 0.015) for Run 5, and 0.033 Hz (SD = 0.033) for Run 6. At 4 Hz, the mean RMSE (SD) was 0.056 Hz (SD = 0.040) for Run 1, 0.062 Hz (SD = 0.050) for Run 2, 0.061 Hz (SD = 0.051) for Run 5, and 0.065 Hz (SD = 0.057) for Run 6. A 4 (run: 1, 2, 5, 6) × 2 (speed: 1 Hz vs. 4 Hz) repeated measures ANOVA of RMSE indicated lower error rates at 1 Hz than 4 Hz, *F*(1, 15) = 8.15, *p* = 0.012. There was no main effect for runs, *F*(1.83, 27.5) = 1.42, *p* = 0.258 and no interaction of speed × run, *F*(1.67, 25.1) = 3.40, *p* = 0.057 (Figure 2). Prolonged periods of inactivity were minimal. At 1 Hz, the median (IQR) of missed taps was 1.1% (0–2.2%) in Run 1; 0% (0–1.1%) in Run 2; 1.1% (0–2.2%) in Run 5; and 1.1% (0–2.2%) in Run 6. At 4 Hz, the median (IQR) of missed taps was 0.83% (0.56–1.4%) in Run 1; 0.56% (0.56–1.4%) in Run 2; 0.56% (0.56–1.7%) in Run 5; and 0.28% (0.28–1.6%) in Run 6.

#### 3.1.2. Imagery Task

ITAP accuracy on Run 3 was 57.4% (SD = 20.8%) and on Run 4 was 55.1% (SD = 11.9%). noITAP accuracy on Run 3 was 59.1% (SD = 15.3%) and on Run 4 was 57.3% (SD = 16.5%). A 2 (run: 3 and 4) × 2 (trial type: ITAP vs. noITAP) repeated measures ANOVA of accuracy indicated no main effect of run *F*(1, 15) = 6.53, *p* = 0.432, no main effect of trial type, *F*(1, 15) = 0.113, *p* = 0.742 and no interaction of run × trial type, *F*(1, 15) = 0.005, *p* = 0.944. One-sample *t*-tests compared overall imagery accuracy for Run 3 (M = 58.3%, SD =10.7%) and Run 4 (M = 56.2%, SD = 8.5%) to a hypothesized chance value of 50%, which revealed statistically significant differences in above-chance accuracy on both runs, t(15) = 3.100 and 2.931, *p* = 0.007 and 0.010, respectively (Figure 2).

#### 3.1.3. Post RtfMRI-NF Tapping Improvements

**Figure 3:**
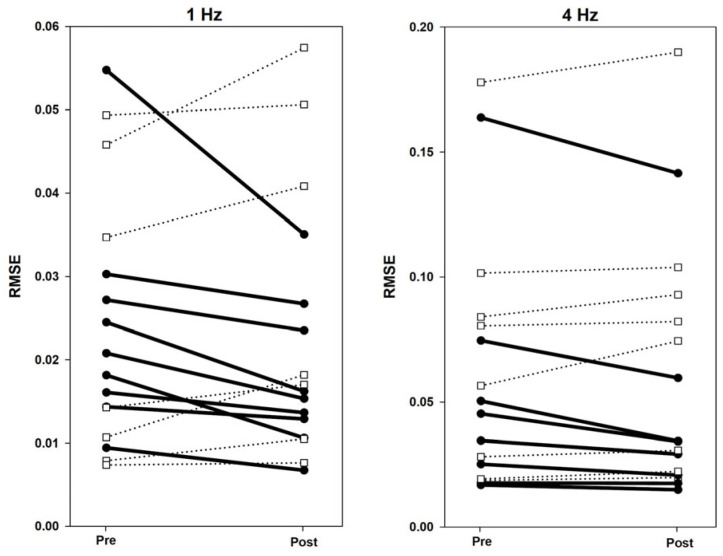
Individual Δ RMSE values from pre- to post-imagery for 1 Hz and 4 Hz. Bold lines indicate task improvement by lowering RMSE post-imagery. Dotted lines indicate no task improvement post-imagery.

Tapping improvements were computed as the Δ RMSE between runs before and just after the imagery neurofeedback training blocks at both tapping speeds. RMSE differences between Tapping Runs 2 vs. 5 were not significant at 1 Hz (*Z* = −0.776, *p* = 0.438) or 4 Hz (*Z* = −0.414, *p* = 0.679). However, a goal of this study was to examine individual effects from the neurofeedback, given the heterogeneity of CA severities and subject variability in performing the motor imagery task. As shown in Figure 3, Δ RMSE decreased (improved) between blocks for 9/16 participants. However, improvement may have resulted from motor learning and/or following a motor “break” during the imagery sessions, without particular influence from the neurofeedback training. To ascertain the effects of neurofeedback training on motor performance, Δ RMSE values were entered as a covariate during the fMRI successful motor imagery analyses in SPM12 to identify brain regions where changes in activity level correlated with tapping accuracy improvement (reported in Section 3.5).

### 3.2. Neural Targets Defined by the Trained SVM Models

**Figure 4:**
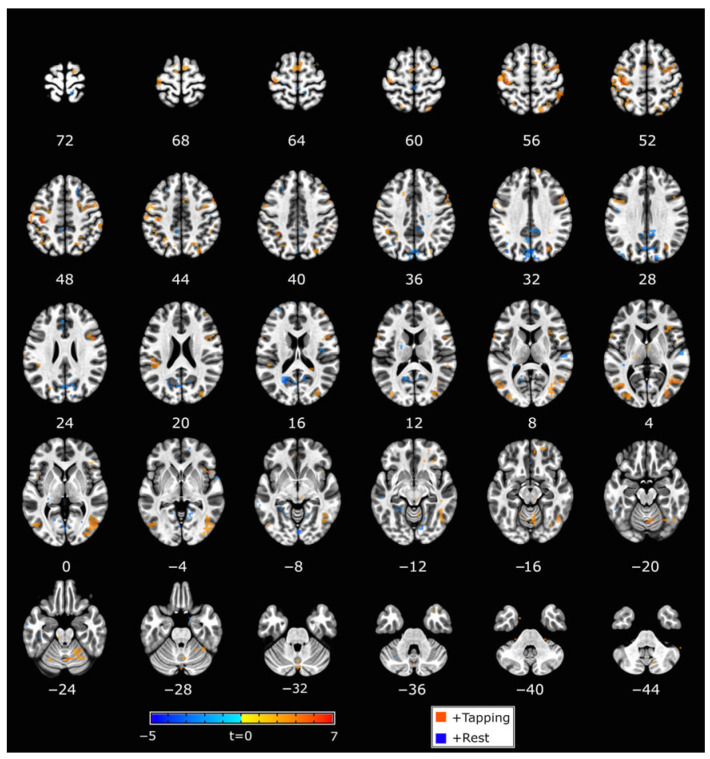
Group classifier weight map. Clusters shown survived the *p* < 0.005 (t = 3.1) threshold and volume > 40 voxels. Red voxels are associated with tapping, and blue voxels are associated with rest. The left side of the brain is shown on the left.

Individualized SVM models (weight vector maps) were created based on finger tapping, including a motor circuit (e.g., primary motor cortex, premotor cortex, and cerebellum). Although a mask of the primary visual cortex was used during the model training step, a group map of the average classifier weights indicated that other visually related brain regions were also important for the classifier. This included the frontal eye fields and visual association areas (Figure 4). The group mask exemplifies the average neural targets of the neurofeedback training, though the weight vector maps used to control neurofeedback varied at the individual level.

As a positive control, we verified that the classifier was accurate and stable by examining classification accuracy on Runs 5 and 6. Classification accuracy for prediction of tapping blocks in Runs 5 and 6 was 68.8% (SD = 11.5%). Accuracy for prediction of rest blocks was 66.9% (SD = 8.8%). Overall accuracy was 67.8% (SD = 8.7%) and, based on a one-sample *t*-test compared to a hypothesized chance value of 50%, performance was significantly above chance, t(15) = 7.43, *p* = 0.00000211.

### 3.3. FMRI Tapping Task

Tapping task (Runs 1, 2, 5, 6) activations were collapsed across speeds. Large activation clusters included visual cortex regions and extended into the primary somatosensory cortex, supplementary motor area (SMA), anterior insula, and prefrontal cortex. Superior and inferior cerebellar activity contained peak clusters on the left side and extended bilaterally (See Table S2).

### 3.4. FMRI Successful Motor Imagery

**Figure 5:**
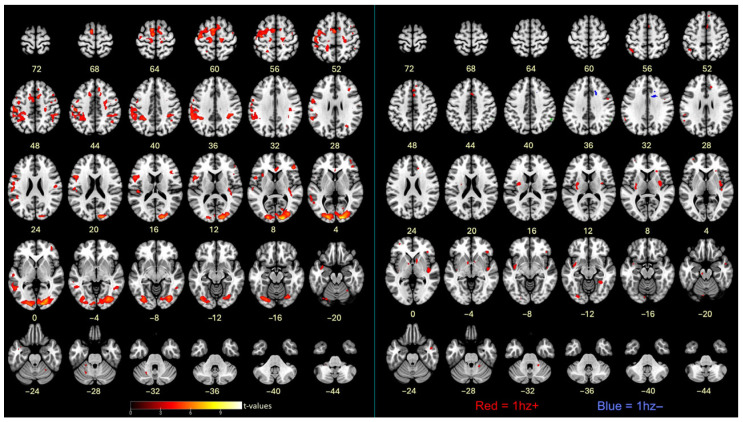
(**left**) Contrasts between successful motor imagery vs. all other trials. (**right**) Covariates of Δ RMSE tapping accuracy (improvements) with successful motor imagery; cluster colors reflect correlations between cluster activation level and tapping accuracy improvements. Red = positive correlation; blue = negative correlation; *p* < 0.001. The left side of the brain is shown on the left. See Methods for cluster thresholds.

Using an event-related analysis, successful motor imagery events (i.e., true ITAP TRs) were compared to all other events in Runs 3 and 4, as shown in Figure 5 and Table 2. The most robust activations were located in visual, frontal, supplementary, and premotor brain regions. Cerebellar regions were also activated, mainly on the left side.

### 3.5. FMRI Successful Imagery Covariates

**Figure 6:**
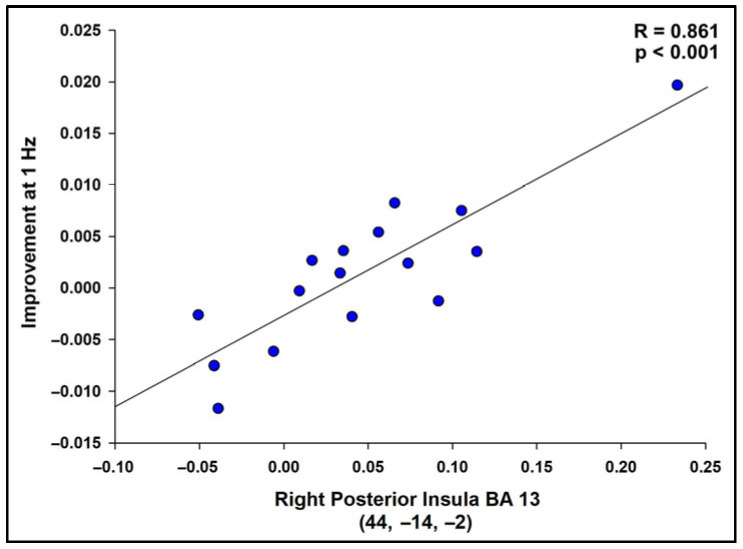
Scatterplot displaying the correlation between successful imagery BOLD activation and improvement in the tapping task at 1 Hz (RMSE) for the region with the highest t-value, R = 0.861, *p* < 0.001. Scatterplots were similarly strong for all regions in Table 3, indicating that the results were not driven by a small number of participants. See Table S3 for correlation values.

**Figure 7:**
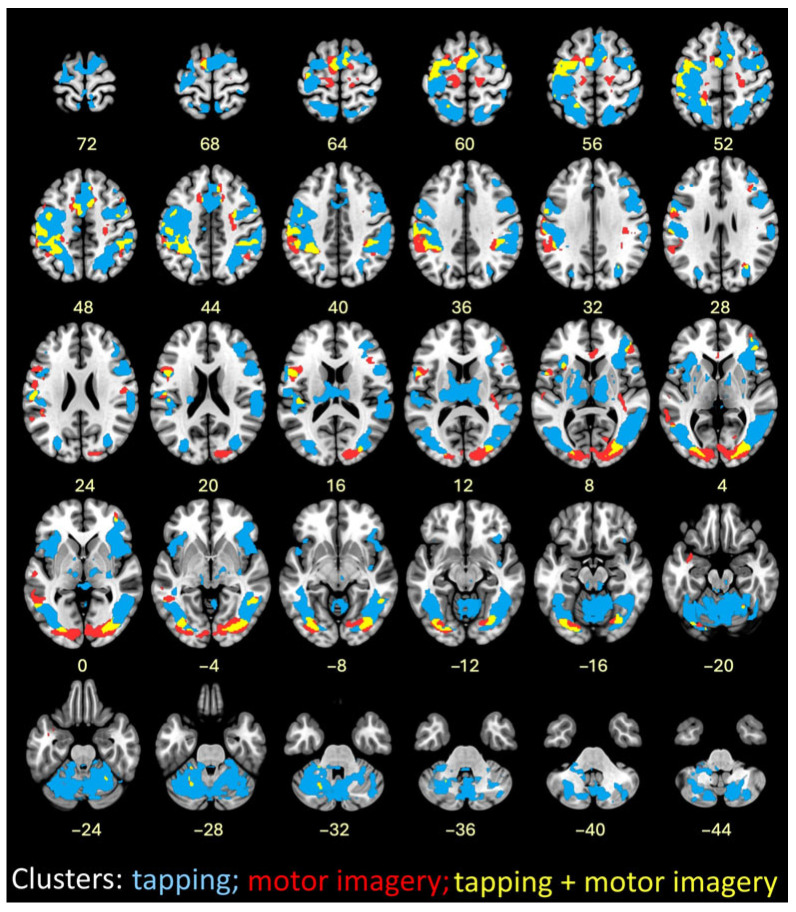
Image overlay of finger tapping at both speeds combined (blue); successful motor imagery (red); and conjunction of finger tapping and successful motor imagery (yellow).

Successful motor imagery events covaried with individual tapping accuracy improvement scores (Δ RMSE values), as shown in Figure 5 and Table 3. Most covariates were significant and positive at the 1 Hz speed, indicating that larger improvements in tapping accuracy were associated with increasing brain activations during successful motor imagery. These were located in the frontal lobe, insula, parietal lobe, basal ganglia, and cerebellum. One activation, located in the frontal lobe white matter, negatively covaried with improvement at 1 Hz (Figure 5 and Table 3). At 4 Hz, the angular gyrus positively covaried with improvement (Table 3). Visualization of scatterplots indicated strong associations that were not driven by a few individuals (Figure 6 and Table S3). A conjunction analysis showed overlap between tapping and imagery in frontal (including SMA, premotor, and sensorimotor), parietal, visual, and cerebellar regions (Figure 7).

### 3.6. Clinical and Cognitive Correlates

Higher motor impairment severity, as measured by ICARS scores, correlated with lower tapping accuracy (RMSE) at 1 Hz on Runs 2, 5, and 6 (r = 0.780, 0.833, and 0.516, respectively) and at 4 Hz on Runs 1, 2, 5, and 6 (r = 0.812, 0.834, 0.779, and 0.846, respectively). ICARS scores did not correlate with delta tapping improvement at either speed (1 Hz, r = −0.145; 4 Hz, r = 0.212). Similarly, delta tapping improvements did not correlate with assessments of mood, cognition, or imagery vividness.

## 4. Discussion

In this study, the feasibility of motor imagery as a form of rehabilitation was tested for those with CA. Participants completed finger tapping and motor imagery tasks while receiving rt-fMRI-NF. Feedback coincided with motor imagery and was designed to steer participants toward the activation of motor neural pathways. It was hypothesized that motor imagery “exercise” of motor neural pathways would improve motor coordination on post-intervention tapping blocks. While participants did not show significantly higher tapping accuracy post-intervention at the group level, greater brain activation in the frontal lobe, insula, basal ganglia, and cerebellum during rt-FMRI-NF-guided motor imagery correlated with larger improvements in accuracy at 1 Hz across participants, suggesting that imagery-related activity contributed to those improvements.

Neuroimaging studies of motor imagery (without feedback) in healthy participants have reported activity consistent with brain regions observed in the current study, which demonstrates intact motor imagery mechanisms in CA, in contrast to their overt motor impairments [19]. The benefit of using rt-fMRI-NF is to optimally explore motor imagery’s therapeutic potential while simultaneously addressing a critical need to better delineate motor control and imagery circuitry in CA. Only a handful of rt-fMRI-NF studies have been conducted in neurologic conditions thus far. In three small stroke studies, participants (N = 15 total) were able to modulate brain networks to improve motor and visuomotor function [30,65,66,67]. In Parkinson’s disease (PD), Tinaz et al. (2022) [68] found that connectivity strength between the insula and dorsomedial frontal cortex correlated with changes in motor function scores in the group that received rt-fMRI-NF (n = 22) but not in the control (non-NF) PD group (n = 22). Interestingly, despite this association, controlled regulation of the insula-dorsomedial frontal cortex was not achieved [68]. A study by Subramanian et al. (2016) [69] compared at-home motor training with (n = 13) and without (n = 13) rt-fMRI-NF, targeting the SMA in PD participants. Improvements in the rt-fMRI-NF group were two-fold greater than in the exercise-only group (who did not show a clinically relevant change), yet this group difference did not reach statistical significance [69]. It is notable that PD participants were guided to specifically engage their insula-dorsomedial frontal circuit and SMA, respectively, between the two research studies. Our agnostic, whole-brain classifier approach allowed the model to train on the entire finger tapping neural network and was individualized across CA participants, which may have been advantageous for optimizing the impact of neurofeedback on motor improvement with a focus on larger networks rather than specific pathways or regions that potentially have differing activations across individuals.

Notably, the insula was found to have the strongest relation between successful motor imagery and motor improvements. This region has been previously observed when individuals successfully engage in neurofeedback [44]. The insula plays a significant role in interoception and somatosensory awareness, which are ways in which body awareness is achieved [70,71,72,73]. Being able to sense the internal state of one’s body is necessary for planning and controlling voluntary movements, and also providing representational information so that movements can be imagined [74]. If interoception was intensified through increased insula activity, a greater sense of the tapping pathway could have been achieved, enhancing tapping performance. Given that cerebellar automaticity of motor functions could be damaged in CA, it is unsurprising that CA participants might rely on interoceptive processes as they attempted explicit strategies of motor imagery [55,75]. Thus, while insula function may not be a typical part of the frank motor circuit, it likely played an important part in successful motor imagery in CA.

The majority of improvements took place in the 1 Hz tapping blocks, with 4 Hz tapping performance showing little to no improvement. It is likely that the 4 Hz speed was too fast for the CA participants to manage, as evidenced by the higher error rate associated with 4 Hz than 1 Hz tapping rates. Previous research indicates different brain activation patterns between slower and faster motions, with slower movements associated with orbitofrontal, prefrontal, and basal ganglia regions, and fast movements associated with sensorimotor and anterior vermian cerebellar regions [76,77]. The characteristic cerebellar degeneration in CA, therefore, might be responsible for the failure of participants to improve their 4 Hz tapping performance: the infrastructure to tap faster and in a more automated fashion was disrupted. Interestingly, the cerebellum (Lobules V and VIIIB) was a covariate for motor improvement at 1 Hz. Thus, as the cerebellum was able to engage, it appeared to benefit from motor imagery to improve motor function. The frontal lobe and basal ganglia regions, involved in slower and more controlled movements, may have been relatively intact in some CA participants, providing an alternative neural basis that allowed motor imagery to improve motor function.

There were several limitations to this feasibility study. First, not all of the participants in this study increased brain activation or experienced motor improvement. These outcomes were unrelated to measures of neurological impairment, mood, cognition, and imagery vividness. However, it is possible that differences in performance were related to neural degeneration outside of the cerebellum, which varies by CA subtype and could not be addressed in the current study design [56,78,79,80]. The heterogeneity of CA across subtypes may help explain why motor improvements were observed in some participants but not others. Further research is needed to clarify which underlying characteristics created barriers and which were most beneficial to motor imagery and motor improvement within the context of CA. Second, within this study design, it is possible that brain activations during imagery runs were not attributable to imagery alone. For example, activations may have been related to small, unintentional overt movements that were not seen by the experimenters. Alternative explanations for the observed improvements include a placebo effect, learning consolidation during imagery runs, or the resumption of tapping after a “rest break” of the imagery runs. Comparison to a control group is warranted to clarify the potential influence of these factors. Third, this was a relatively small sample size that did not examine a priori ROIs, and this required a somewhat lenient statistical threshold (*p* < 0.001 without multiple comparison corrections). While this increased the risk of type I errors, we opted for this broader, inclusive approach given that CA represents a rare neurological disease in which this type of investigation had never been attempted. Finally, it is impossible to know what participants were truly thinking during motor imagery. Thus, neurofeedback could have, at times, reinforced incorrect strategies or failed to reinforce strategies that were correct but used at the wrong time, which could have hindered learning for some people. We are unable to disentangle those relationships in the current study design.

The characterization of brain activity findings in this study helps establish target neural mechanisms for treatment. For instance, non-invasive brain stimulation methods like transcranial direct current stimulation (tDCS) could be applied to superficially located brain regions during motor imagery to improve motor outcomes, as demonstrated in young adults and stroke patients [81,82]. Importantly, these preliminary results suggest feasibility of using motor imagery in a therapeutic context to investigate potential motor-function improvements in CA. Moreover, these results lay the foundation for motor imagery methods, with refinement, to be scaled to explore possible improvements of more complex movements, including limb and gait function, to improve the quality of life for those living with CA and other movement disorders.

## 5. Conclusions

This study explored the feasibility of combining motor imagery with rt-fMRI-NF to improve motor function in individuals with neurodegenerative CA. Although group-level finger tapping accuracy improvements were not significant, improvements were observed at the individual level. Greater brain activation in key regions during successful imagery was correlated with better motor performance, particularly at 1 Hz tapping rates. However, results should be interpreted within the context of the study design, which included limitations, such as heterogeneity of CA subtypes, a small sample size, and lack of a control group. Nonetheless, these preliminary findings suggest that motor imagery combined with neurofeedback is a feasible approach to potentially enhance fine motor skills in CA.

## Figures and Tables

**Table 1:** Demographic variables of the CA participants.

Demographic Variables	Values (SD)
M:F	7:9
Age in Years (SD)	57.94 (13.1)
Education in Years (SD)	16.25 (2.1)
Duration of Illness in Years (SD)	14.13 (11.4)
**Number of participants per CA Subtype**	
SCA2	1
SCA3	4
SCA5	1
SCA6	2
SCA8	3
SCA27B	1
SCA34	1
SCA50	1
CAUE	2
**Clinical Variables**	**Values (SD)**
HAM-A (SD)	5.50 (5.1)
HAM-D (SD)	3.06 (2.8)
WRAT4 (SD)	47.75 (3.8)
CCAS (SD)	94.63 (14.1)
KVIQ-10 (SD)	37.44 (10.1)
AAF (SD)	32.69 (15.9)
ICARS (SD)	32.38 (19.2)

M = male; F = female; SD = standard deviation; CAUE = cerebellar ataxia of unknown etiology. HAM-A = Hamilton Anxiety Scale; HAM-D = Hamilton Depression Rating Scale; WRAT4 = Wide-Range Achievement Test 4; KVIQ-10 = Kinesthetic and Visual Imagery Questionnaire 10; CCAS = Cerebellar Cognitive-Affective Syndrome Scale; AAF = Ataxia Assessment of Function; ICARS = International Cooperative Ataxia Rating Scale.

**Table 2:** Activation table of brain regions associated with successful motor imagery.

Successful Motor Imagery			
Cluster Size (Voxels)	t-Value	X, Y, Z (MNI)	Brain Region (BA)
2867	11.14	12, −94, 2	R Primary Visual Cortex (17) *
3261	7.00	−6, −8, 62	L Supplementary Motor Area (6)
83	5.86	46, 34, 8	R Inferior Frontal Gyrus (45)
326	5.48	−50, 14, 22	L Inferior Frontal Gyrus (44)
193	5.40	−54, −48, −2	L Middle Temporal Gyrus (21)
300	5.38	12, −22, 60	R Middle Frontal Gyrus (6)
353	5.33	36, −36, 38	R Inferior Parietal Lobe (40)
36	5.32	10, 28, 46	R Superior Frontal Gyrus (8)
52	5.04	6, 32, 10	R Anterior Cingulate (24)
23	4.88	54, 0, 46	R Precentral Gyrus (6)
77	4.86	36, −20, 10	R Posterior Insula (13)
44	4.77	40, 54, 2	R Middle Frontal Gyrus (10)
37	4.73	48, 12, 50	R Middle Frontal Gyrus (8)
29	4.72	28, −64, 28	R Superior Temporal Gyrus (39)
19	4.69	44, −6, 60	R Precentral Gyrus (6)
21	4.63	36, 30, 28	R Middle Frontal Gyrus (9)
33	4.61	−36, 4, −22	L Superior Temporal Gyrus (38)
73	4.58	52, −50, −6	R Middle Temporal Gyrus (21)
40	4.52	50, −16, 24	R Post Central Gyrus (BA 1)
40	4.51	−24, −60, −29	L Cerebellar Lobule VI
44	4.41	-58, −16, 0	L Superior Temporal Gyrus (22)
16	4.36	−22, −40, −47	L Cerebellar Lobule VIIIB
44	4.35	38, −22, 32	R Supramarginal Gyrus (40)
16	4.33	28, −56, −19	R Cerebellar Lobule VI
20	4.28	42, −6, 50	R Precentral Gyrus (6)
23	4.27	−32, 16, 8	L Anterior Insula (13)
29	4.14	46, 20, 14	R Inferior Frontal Gyrus (44)
12	4.00	−24, −42, −27	L Cerebellar Lobule V

Successful imagery events were isolated relative to all other events in Runs 3 and 4. * Note that BA 17 was included in the visual cortex mask that excluded primary visual areas from the SVM model. It is reported here as a region that was sensitive to the flashing cross, but it was not used as part of the classifier.

**Table 3:** Activation table of brain regions associated with successful motor imagery and tapping improvements.

Successful Motor Imagery Covaried with Tapping Improvements (Δ RMSE)			
Cluster Size (Voxels)	t-Value	X, Y, Z (MNI)	Brain Region (BA)
1 Hz (+) Covariates			
223	7.70	44, −14, −2	R Posterior Insula (13)
50	7.65	44, 32, −6	R Inferior Frontal Gyrus (47)
52	7.58	−44, −54, 54	L Angular Gyrus (39)
26	7.32	22, −44, −31	R Cerebellar Lobule V
87	7.22	−40, −8, −8	L Posterior Insula (13)
124	6.78	−26, −8, 16	L Putamen
28	6.57	10, 8, −2	R Caudate
89	6.25	4, 12, 46	R Supplementary Motor Area (6)
23	6.08	−10, 4, −2	L Globus Pallidus
38	6.03	−28, −84, −12	L Inferior Occipital Gyrus (18)
23	5.65	12, 34, 26	R Dorsal Anterior Cingulate (32)
36	5.60	−58, −52, 34	L Supramarginal Gyrus (39)
26	5.25	46, −2, −24	R Middle Temporal Gyrus (21)
21	5.23	24, 24, 8	Right Caudate/White Matter
15	5.17	8, 34, 50	R Superior Frontal Gyrus (8)
21	5.12	−34, 56, 2	L Middle Frontal Gyrus (10)
11	4.92	−10, −24, −19	L Substantia Nigra
18	4.87	50, 2, 36	R Precentral Gyrus (6)
13	4.66	−24, −40, −55	L Cerebellar Lobule VIIIB
1 Hz (−) Covariates			
61	6.03	20, 14, 36	L Frontal White Matter
4 Hz (+) Covariates			
21	5.14	56, −52, 40	R Angular Gyrus (39)
4 Hz (−) Covariates—none			

Successful motor imagery events were covaried with tapping accuracy improvement (Δ RMSE values).

## Data Availability

The data presented in this study are available on request from the corresponding author. Data are not publicly available due to privacy restrictions.

## Supplementary Materials

The following supporting information can be downloaded at https://www.mdpi.com/article/10.3390/brainsci16020120/s1. Table S1: Demographic data of four excluded CA participants; Table S2: Activations associated with finger tapping; Table S3: Brain regions associated with successful motor imagery that correlated with tapping improvements (Δ RMSE) at 1 Hz; Video S1: Tapping; Video S2: ITAP; Video S3: noITAP.
