# Supplementary material for: A Feasibility Study of Real-Time FMRI with Neurofeedback of Motor Performance in Cerebellar Ataxia
Source: Brain Sci. 2026 Jan 23;16(2):120. doi: 10.3390/brainsci16020120 (PMC12937812; doi:10.3390/brainsci16020120)
Supplement: Supplementary file 1 [file brainsci-16-00120-s001.zip › Table S1 Excluded demo.pdf]

**Table S1. Demographic data of four excluded CA participants.**
Four participants, with subtypes of SCA3, SCA6, SCA8, and CAUE, were excluded from the data. Mann-Whitney t-tests indicated no significant demographic differences between the excluded versus included participants shown in Table 1.

| **Demographic Variable** | **Values (SD)** |
|---|---|
| Males:Females | 2:2 |
| Age (years) | 58.90 (6.98) |
| Education (years) | 17.50 (1.9) |
| Duration of illness (years) | 18.00 (7.9) |
| HAM-A | 4.25 (4.3) |
| HAM-D | 2.25 (1.3) |
| WRAT4 | 50.00 (2.9) |
| CCAS | 96.33 (10.1) |
| KVIQ-10 | 40.25 (7.4) |
| AAF | 37.50 (12.7) |
| ICARS | 45.00 (21.0) |

SD = standard deviation; HAM-A = Hamilton Anxiety Scale; HAM-D = Hamilton Depression Rating Scale; WRAT4 = Wide Range Achievement Test 4; KVIQ-10 = Kinesthetic and Visual Imagery Questionnaire 10; CCAS = Cerebellar Cognitive Affective Syndrome Scale; AAF = Ataxia Assessment of Function; ICARS = International Cooperative Ataxia Rating Scale.

**Reported strategies by excluded participants**
In a post-experimental questionnaire, participants were asked the following:

1) On trials in which you imagined tapping, where you were asked to "make the cross flash", describe your strategy for imagining tapping. *Participants were excluded if responses were not motor-focused or included overt movements.*
2) On trials in which you did not imagine tapping, where you were asked to "make the cross stop", describe your strategy for not imagining tapping. *Participants were excluded if responses were motor-focused or included overt movements.*

Four participants' data were excluded from analyses because responses to #1 and/or #2 were unacceptable.

Subject A
1) "I thought of the word 'Go! Go!' without any visuals" – *Disqualified because the strategy was not motor-focused.*
2) "I visualized my warm fuzzy cat sitting in my lap." -- *Acceptable*

Subject B
1) "I looked at the cross and wished it would flash. I pictured that it would start flashing."-- *Disqualified because the strategy was not motor-focused.*
2) "I thought about the cross, that it would stop." -- *Acceptable*

Subject C
1) "[I imagined using a] joystick with button on top, then changed to piano key, then to drum slamming." -- *Acceptable*
2) "I focused on thinking about doing tapping incorrectly. That worked some of the time." – *Disqualified because the strategy was motor-focused.*

Subject D
1) "Saying tap over and over worked."—*Disqualified because the strategy included overt movements.*
2) "Counted numbers."– *Disqualified because the strategy included overt movements.*
