# Supplementary material for: A Feasibility Study of Real-Time FMRI with Neurofeedback of Motor Performance in Cerebellar Ataxia
Source: Brain Sci. 2026 Jan 23;16(2):120. doi: 10.3390/brainsci16020120 (PMC12937812; doi:10.3390/brainsci16020120)
Supplement: Supplementary file 1 [file brainsci-16-00120-s001.zip › Table S2 Tapping clusters.pdf]

Table S2. Activations associated with finger tapping.

Cluster Size (voxels)	t-value	x, y, z (MNI)	Brain Region (BA)
24,428	11.39	46, -62, -2	R Inferior Temporal Gyrus (19)
8,456	9.74	-32, -68, -29	L Cerebellar Lobule VI
2,883	8.92	-44, -62, -10	L Inferior Temporal Gyrus (19)
66	5.89	-8, -18, 1	L Thalamus
44	5.58	-8, -22, 48	L Cingulate Gyrus (31)
32	5.39	-10, -60, -55	L Cerebellar Lobule IX
26	4.72	-48, 28, 28	L Middle Frontal Gyrus (9)
91	4.69	40, -8, -10	R Posterior Insula (13)
15	4.08	-6, -40, -43	L Pons

Regions of activation for the Tapping condition (all speeds) minus Rest. MNI = Montreal Neurological Institute coordinate system; BA = Brodmann Area.
