# Supplementary material for: A Feasibility Study of Real-Time FMRI with Neurofeedback of Motor Performance in Cerebellar Ataxia
Source: Brain Sci. 2026 Jan 23;16(2):120. doi: 10.3390/brainsci16020120 (PMC12937812; doi:10.3390/brainsci16020120)
Supplement: Supplementary file 1 [file brainsci-16-00120-s001.zip › Table S3 Correlations .pdf]

Table S3. Brain regions associated with successful motor imagery that correlated with tapping improvements (Δ RMSE) at 1 Hz.

X, Y, Z (MNI)	Brain Region (BA)	Pearson Correlation Coefficient between MRI ROI values and Δ RMSE	p -value
44, -14, -2	R Posterior Insula (13)	0.861	< .001
44, 32, -6	R Inferior Frontal Gyrus (47)	0.838	< .001
-44, -54, 54	L Angular Gyrus (39)	0.713	.002
22, -44, -31	R Cerebellar Lobule V	0.831	< .001
-40, -8, -8	L Posterior Insula (13)	0.842	< .001
-26, -8, 16	L Putamen	0.884	< .001
10, 8, -2	R Caudate	0.850	< .001
4, 12, 46	R Supplementary Motor Area (6)	0.786	< .001
-10, 4, -2	L Globus Pallidus	0.718	.002
-28, -84, -12	L Inferior Occipital Gyrus (18)	0.766	< .001
12, 34, 26	R Dorsal Anterior Cingulate (32)	0.799	< .001
-58, -52, 34	L Supramarginal Gyrus (39)	0.811	< .001
46, -2, -24	R Middle Temporal Gyrus (21)	0.694	.003
24, 24, 8	Right Caudate/White Matter	0.694	< .001
8, 34, 50	R Superior Frontal Gyrus (8)	0.802	< .001
-34, 56, 2	L Middle Frontal Gyrus (10)	0.765	< .001
-10, -24, -19	L Substantia Nigra	0.765	.003
50, 2, 36	R Precentral Gyrus (6)	0.740	< .001
-24, -40, -55	L Cerebellar Lobule VIIIB	0.740	< .001
